# Comment on "Predictive Value of Mean Platelet Volume in Saphenous Vein Graft Disease"

**DOI:** 10.21470/1678-9741-2018-0283

**Published:** 2018

**Authors:** Erkan Coban

**Affiliations:** 1 Akdeniz University Faculty of Medicine, Department of Internal Medicine, Antalya, Turkey.

Dear Editor,

I have read with great interest the recently published article by Kaya et al.^[1]^. This study's findings indicated that mean platelet volume (MPV) may be a useful indicator for the prediction of saphenous vein grafts disease (SVGD) and mortality following coronary artery bypass graft (CABG) surgery. However, there are major limitations about MPV levels in this study:

In this study, MPV measurement technique is not written. Pre-analytical variables, such as the anticoagulant used, and the time between blood collection and measurement are known to significantly affect MPV measurements. Although EDTA is traditionally used and recommended for samples destined for blood counting it is well known that platelets collected into EDTA anticoagulants undergo time-dependent platelet swelling and activation^[2,3]^. The retrospective nature of the study leads to a significant problem because the MPV results could not be standardized.

This study is retrospective and its duration is approximately 7 years. However, recent studies have shown seasonal changes in MPV levels^[4]^. On the other hand, different devices and technologies used in MPV measurements can produce deviations^[5]^.

## Disclosure of Interest

We declare that they have no conflicts of interest concerning this article.

The results of the current study differ from an experimental study in South Korea in 2003 which was also performed on rat hearts (!) being protected either by original Custodiol or by Del Nido solution. It became obvious that after two-hour-arrest mitochondrial scoring was superior in the Custodiol group^[5]^.
